# DLP1‐dependent mitochondrial fragmentation and redistribution mediate prion‐associated mitochondrial dysfunction and neuronal death

**DOI:** 10.1111/acel.12693

**Published:** 2017-11-22

**Authors:** Chaosi Li, Di Wang, Wei Wu, Wei Yang, Syed Zahid Ali Shah, Ying Zhao, Yuhan Duan, Lu Wang, Xiangmei Zhou, Deming Zhao, Lifeng Yang

**Affiliations:** ^1^ National Animal Transmissible Spongiform Encephalopathy Laboratory State Key Laboratories for Agrobiotechnology Key Laboratory of Animal Epidemiology and Zoonosis College of Veterinary Medicine Ministry of Agriculture China Agricultural University Beijing 100193 China

**Keywords:** dynamin‐like protein 1, mitochondrial dynamics, neuronal degeneration, prion disease

## Abstract

Mitochondrial malfunction is a universal and critical step in the pathogenesis of many neurodegenerative diseases including prion diseases. Dynamin‐like protein 1 (DLP1) is one of the key regulators of mitochondrial fission. In this study, we investigated the role of DLP1 in mitochondrial fragmentation and dysfunction in neurons using *in vitro* and *in vivo* prion disease models. Mitochondria became fragmented and redistributed from axons to soma, correlated with increased mitochondrial DLP1 expression in murine primary neurons (N2a cells) treated with the prion peptide PrP^106–126^ *in vitro* as well as in prion strain‐infected hamster brain *in vivo*. Suppression of DLP1 expression by DPL1 RNAi inhibited prion‐induced mitochondrial fragmentation and dysfunction (measured by ADP/ATP ratio, mitochondrial membrane potential, and mitochondrial integrity). We also demonstrated that DLP1 RNAi is neuroprotective against prion peptide in N2a cells as shown by improved cell viability and decreased apoptosis markers, caspase 3 induced by PrP^106–126^. On the contrary, overexpression of DLP1 exacerbated mitochondrial dysfunction and cell death. Moreover, inhibition of DLP1 expression ameliorated PrP^106–126^‐induced neurite loss and synaptic abnormalities (i.e., loss of dendritic spine and PSD‐95, a postsynaptic scaffolding protein as a marker of synaptic plasticity) in primary neurons, suggesting that altered DLP1 expression and mitochondrial fragmentation are upstream events that mediate PrP^106–126^‐induced neuron loss and degeneration. Our findings suggest that DLP1‐dependent mitochondrial fragmentation and redistribution plays a pivotal role in PrP^S^ ^c^‐associated mitochondria dysfunction and neuron apoptosis. Inhibition of DLP1 may be a novel and effective strategy in the prevention and treatment of prion diseases.

## Introduction

Prion diseases such as scrapie, bovine spongiform encephalopathies, Creutzfeldt‐Jakob disease, and Kuru are caused by an unconventional and unique pathogen found in various mammalian species (Liberski, 2012). The underlying cause of prion diseases is the accumulation of an abnormal form (PrP^Sc^) of the cellular prion protein (PrP^C^), which is normally found in healthy brain neurons (DeArmond, 2004). It is believed that in vulnerable neurons, PrP^Sc^ aggregates are attributable to the constant conversion of existing PrP^C^ into PrP^Sc^, leading to marked spongiform vacuolation as well as progressive neuronal loss, which are common and critical pathologic features in prion diseases (Prusiner, 1991).

The neurotoxic PrP fragment 106–126 (PrP^106–126^) owns many physiologic properties of PrP^Sc^. Therefore, PrP^106–126^ is widely used as a model for studying PrP^Sc^ neurotoxicity (Forloni *et al*., 1993; Gu *et al*., 2002; Jeong *et al*., 2011). In parallel with PrP^Sc^, further investigation of the mechanism by which PrP^Sc^ results in neuronal death could provide novel insights into therapeutic targets for prion (Zhu *et al*., 2016).

In mammals, neurons are particularly vulnerable to mitochondrial dysfunction because of the high metabolic rate and complex morphology (Sebastian *et al*., 2017). More specifically, mitochondria are of vital importance for neuronal synaptic development, plasticity, and loss (Flippo & Strack, 2017). During cell proliferation and development, mitochondria provide sufficient energy and undergo continual fission and fusion events mediated by GTPases, such as dynamin‐like protein 1 (DLP1) for fission and mitofusins (MFNs) for fusion (Bertholet *et al*., 2016). Fis1 and DLP1 are both mitochondrial fission proteins but Fis1 primarily plays a role in transporting DLP1 from cytoplasm to mitochondrial surface before the oligomerized DLP1 forms a ring which constricts the organelle and eventually leads to fission (Yoon *et al*., 2003). DLP1 is ubiquitously expressed in mammals and evolutionarily conserved in all eukaryotes. Numerous data showed the crucial role of DLP1 not only in synaptic plasticity but also for neuronal death through DLP1's effects on mitochondrial morphology (Ko *et al*., 2016; Li *et al*., 2016; Wang *et al*., 2016). DLP1‐null mice display embryonic defects in neuronal development associated with dramatic decreases in ATP production and impaired apoptosis (Wang *et al*., 2009). In addition, postnatal deletion of DLP1 in the cerebellum results in gradual neurodegeneration over 4–6 months (Kageyama *et al*., 2012). It is thus not surprising that DLP1 is a very important factor in maintaining the integrity of the nervous system.

Because of the vital role of mitochondria in neurons, deficient mitochondrial dynamics are believed to be a culprit for neurodegenerative diseases (Bertholet *et al*., 2016; Li *et al*., 2016; Yang *et al*., 2016). In Alzheimer's disease (AD) brains, increased level of mitochondrial DLP1 is a primary factor for altered balance in mitochondrial division and fusion, which is likely an important mechanism leading to synaptic loss and neural dysfunction (Wang *et al*., 2009). In Huntington's disease (HD), aberrant mitochondrial function has been postulated to critically contribute to striatal degeneration, and inhibitor of DLP1 corrected defects in mitochondrial function and reduced cell death in multiple HD cell models derived from either mice or patients (Guo *et al*., 2013). Malfunctioned mitochondrial dynamics were detected in scrapie‐infected mice (Choi *et al*., 2014; Yang *et al*., 2016). However, to date, the mechanism of mitochondrial dysfunction associated with PrP^Sc^ remains elusive. In the current study, we investigated the changes of mitochondrial morphology and function and the role of DLP1 in the disruption of mitochondrial dynamic balance and neuron apoptosis induced by PrP^Sc^. Our results showed that in PrP^106–126^‐treated N2a cells and prion‐infected hamster brains, mitochondria undergo excessive fission, correlated with the increased mitochondrial DLP1. Blocking DLP1‐dependent mitochondria fission alleviated PrP^106–126^‐induced mitochondrial damage and neuron apoptosis. Our findings of discovering another possible therapeutic target of prion diseases have significant implications for prion diseases.

## Results

### Mitochondrial fragmentation and redistribution in prion diseases

**Figure 1:**
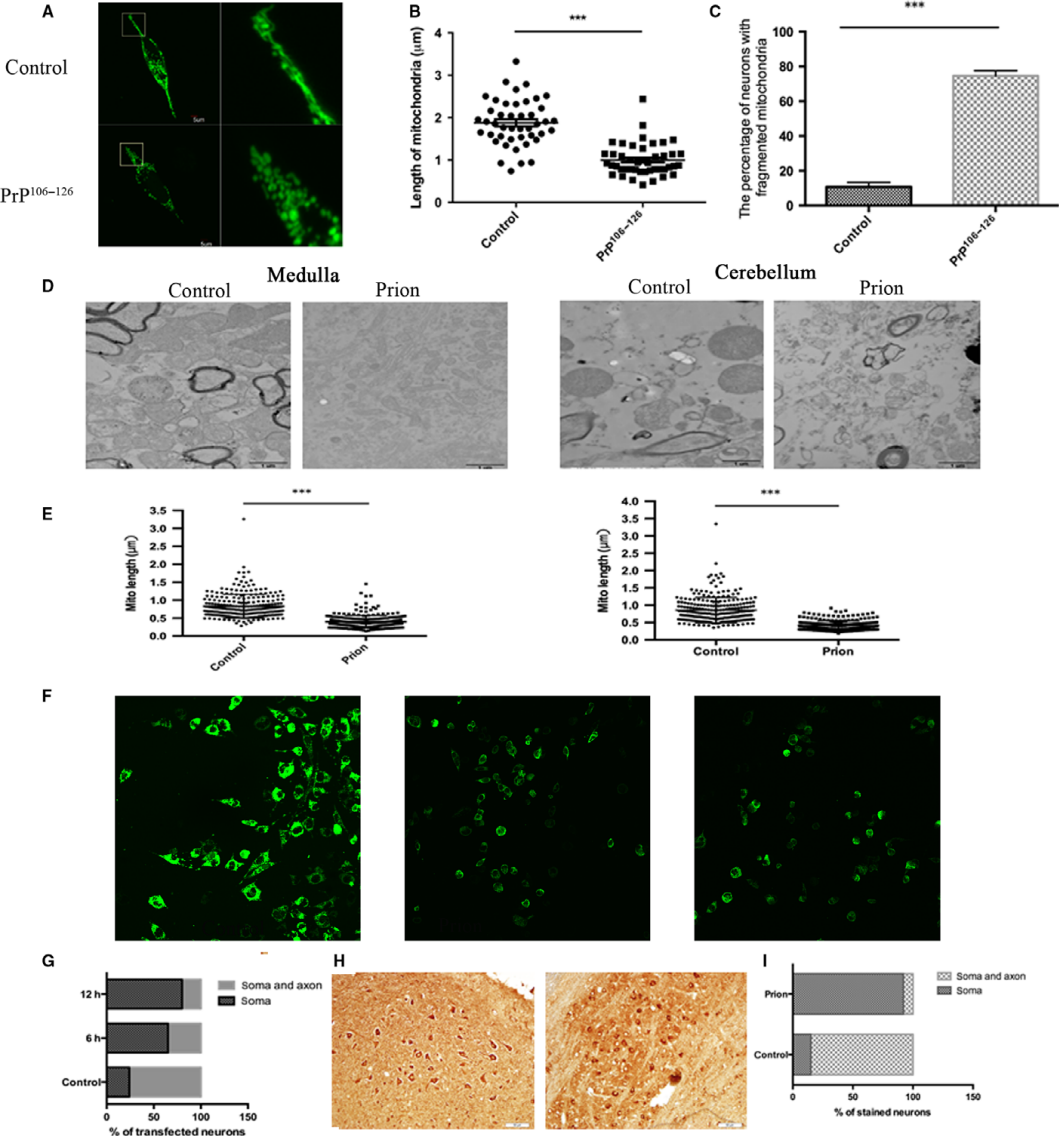
Mitochondrial fragmentation and redistribution in PrP^106–126^‐treated N2a cells and neuronal cells of prion‐infected hamsters. (A) Immunofluorescence image showing a fragmented mitochondrion in PrP^106–126^‐treated N2a cells, compared with a normal mitochondrion in the control cell. (B) Mitochondria length in the prion peptide‐treated cells was significantly shorter than that in the control cells. (C) PrP^106–126^ treatment increased the number of N2a cells with fragmented mitochondria. (D) TEM images showing mitochondrial damage and fragmentation in medulla and cerebellum of 263K scrapie‐infected hamsters. (E) Mitochondria were significantly shorter in scrapie‐infected hamster medulla and cerebellum than in normal hamster brain sections. (F, G) Indirect immunofluorescence images of N2a cells transfected with Mito‐GFP and treated with PrP^106–126^. After incubation with PrP^106–126^ for 6 or 12 h, mitochondria were significantly constricted in soma, while in normal control cells mitochondria were evenly distributed in the soma and axon. (H, I) Immunochemistry using anti‐COX IV antibody illustrated that in prion hamster medulla COX IV staining in neuronal processes was significantly decreased compared with age‐matched controls. All experiments were repeated at least three times. ***P < 0.001.

To investigate whether mitochondrial dynamics was changed in prion diseases like other neurodegenerative diseases, N2a cells were transfected with a green fluorescent protein, namely mito‐GFP specially targeting to the mitochondrial matrix. Thirty‐six hours after transfection, the cells were treated with 150 μm PrP^106–126^ for 12 h and then fixed for examination by fluorescent microscopy. Compared with typical filamentous and tubular mitochondria of untreated cells, PrP ^106–126^‐treated cells showed severe mitochondrial fragmentation illustrated by small, round fragments (Fig. 1A). The mitochondria length was significantly decreased in the prion peptide‐treated cells, compared with the control cells (Fig. 1B). In addition, the percent of cells with fragmented mitochondria was significantly increased to approximately 80% compared with <15% in the untreated control group (Fig. 1C). Sections of cerebellum,medulla,thalamus,hippocampus, and cerebral cortex of 263K prion strain‐infected hamsters were examined under TEM, and 100 mitochondria in each section were randomly counted and mitochondrial length was measured. Mitochondrial length of all brain sections was in varying degrees shorter than that in the control hamster brain. Mitochondria in medulla and cerebellum were significantly smaller than those in the other three regions. Additionally, the damaged and incomplete cristae were clearly observed in the 263K strain‐infected brain sections (Fig. 1D,E). The above findings demonstrate mitochondrial damage and fragmentation *in vitro* and in prion‐infected hamsters.

We also investigated mitochondrial distribution, which is another aspect of mitochondrial dynamics. To determine the distribution of mitochondria in neuronal cells, mito‐GFP and cytochrome C oxidase IV (COX IV) were used as mitochondrial markers. Immunofluorescence imaging revealed considerable redistribution of mito‐GFP from axons to neuronal soma in PrP^106–126^‐treated N2a cells (Fig. 1F,G). Mitochondria in normal cells were evenly distributed in soma and processes of more than 75% cells (>75%). In PrP^106–126^‐treated cells, mitochondria were significantly reduced in neuron processes, and by 12 h after treatment, more than 75% of neuron cells had mitochondria distributed only in cell soma. Similar findings were observed in the brain of 263K strain‐infected hamsters (Fig. 1H,I). Quantitative analysis of COX IV staining in medulla of brain showed a dramatic difference in the percentage of neurons with soma staining only and staining in both soma and processes between prion and age‐paralleled control sections. In negative controls, mitochondria were distributed in soma and processes of most neurons (>80%). In comparison, mitochondria were limited to the soma in >90% of neurons in medulla of 263K strain‐infected hamsters. The above findings confirmed that mitochondria redistributed from neuronal processes and accumulated in the soma of neurons in prion‐infected brain.

Overall, fluorescence imaging, electron microscopy and immunohistochemistry examination of PrP^106–126^‐treated murine neuronal cells and scrapie‐infected hamster brain showed mitochondrial fragmentation and redistribution of mitochondria from neuronal processes to soma, suggesting alteration of mitochondrial dynamics in prion disease.

### Increased mitochondrial DLP1 in prion diseases

**Figure 2:**
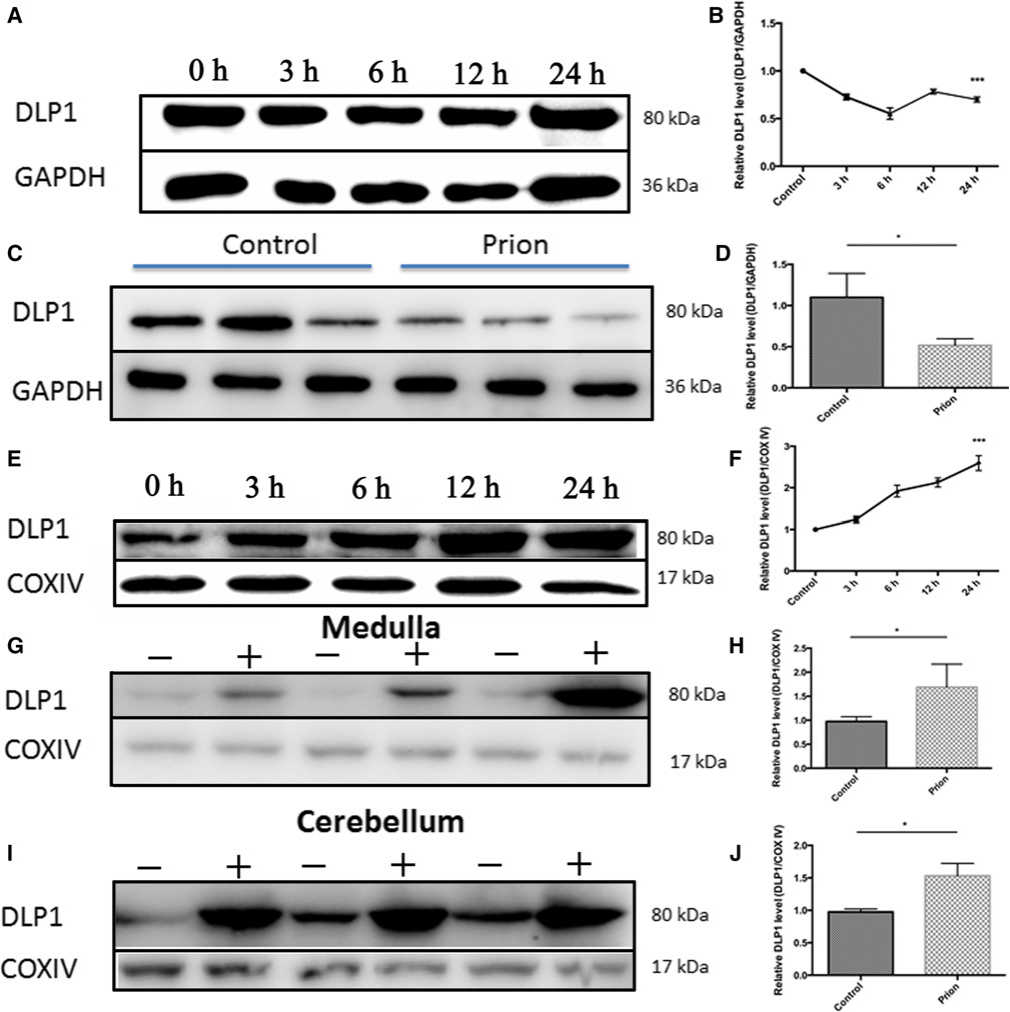
Decreased cellular DLP1 expression and increased mitochondrial DLP1 in prion disease models. Western blotting showed that DLP1 protein decreased in N2a cells treated with 150 μm PrP^106–126^ (A, B) and in 263K strain‐infected hamster brain (C, D). In contrast, the level of mitochondrial DLP1 in PrP^106–126^‐treated N2a cells increased time dependently over the 24‐h exposure period (E, F) and in the brain medulla and cerebellum of hamsters infected with prion (G‐J). Ten prion hamsters and ten control hamsters were analyzed. All *in vitro* experiments were repeated at least three times. *P < 0.05, ***P < 0.001.

Accumulating clinical and experimental evidence indicates that continuous mitochondrial fission leads to excessive mitochondria fragmentation and mitochondrial damage. DLP1 is a critical fission factor responsible for mitochondria division. To determine whether DLP1 is involved in mitochondrial fragmentation in prion disease, we first measured the expression level of DLP1 in PrP^106–126^‐treated N2a cells. DLP1 expression decreased from 3 h after PrP^106–126^ treatment and remained low for the 24‐h observation period (Fig. 2A,B). The level of DLP1 in brain homogenate of hamsters in late‐stage prion disease was also decreased (Fig. 2C,D). Thus, DLP1 was decreased in both the *in vitro* model of prion disease and *in vivo* in the hamster prion disease model.

Most DLP1 resides in the cellular cytoplasm and is transported to mitochondrial outer membranes for mitochondrial fission. We determined the level of DLP1 in mitochondria isolated from PrP^106–126^‐treated N2a cells and the brain of hamsters infected with prion. Mitochondria lysate was subjected to Western blotting for DLP1 and COX IV proteins using appropriate antibodies. Compared with the untreated cells, mitochondrial DLP1 rapidly increased after PrP^106–126^ treatment (Fig. 2E,F). Similarly, the level of mitochondrial DLP1 in medulla and cerebellum also significantly increased (Fig. 2G–J) but did not achieve statistical significance in the other three regions (data not shown).

The above results indicate that the cellular DLP1 protein level decreased in prion‐infected neuronal cells *in vitro* and *in vivo*, but the mitochondrial DLP1 level increased in these prion models.

### DLP1 is involved in PrP^106–126^‐induced mitochondrial fragmentation

**Figure 3:**
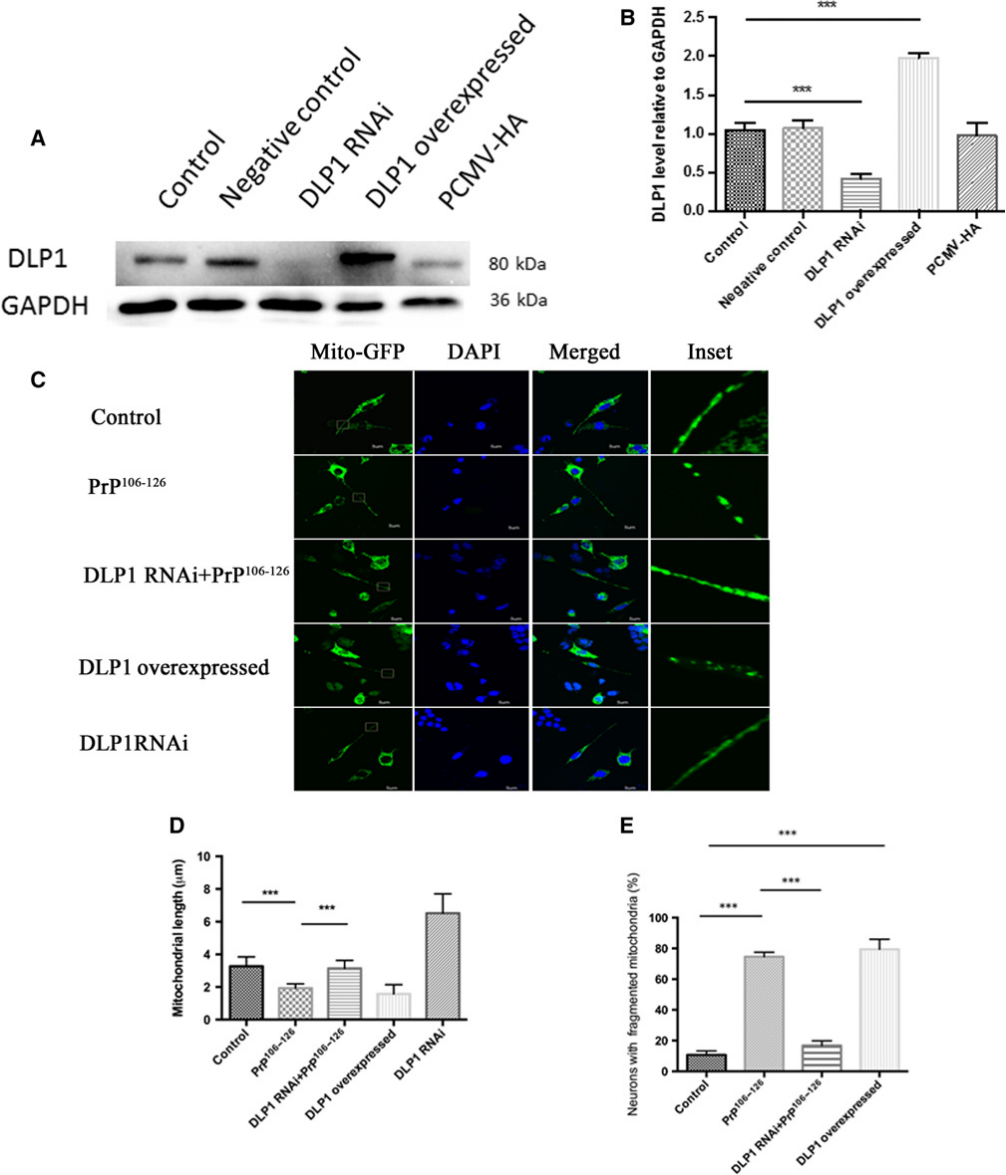
DLP1 is involved in PrP^106–126^‐induced mitochondrial fragmentation. N2a cells were transiently transfected with DLP1 RNAi, PCMV‐DLP1, or a scramble sequence (negative control) together with mito‐GFP. Immunoblotting showed a decrease in DLP1 expression in RNAi‐transfected N2a cells and an increase in DLP1 level in DLP1 overexpressed N2a cells (A, B). N2a cells were co‐transfected with Mito‐GFP and DLP1 RNAi or PCMV‐DLP1, and then treated with 150 μm PrP^106–126^, fixed and analyzed by immunofluorescence imaging. Suppressed DLP1 expression by DLP1 RNAi transfection prevented PrP106‐126‐induced mitochondria fragmentation as shown by immunofluorescence images (C), mitochondria length (D), and percent of cells with fragmented mitochondria (E). Control: wild‐type cells; negative control: cells transfected with scrambled RNAi. *P < 0.05, **P < 0.01, ***P < 0.001.

Based on the above results, we suspected that DPL1 may be involved in mitochondrial damage in prion diseases. N2a cells were transiently transfected with either DLP1 RNAi or a scramble sequence as the negative control together with mito‐GFP and then treated with PrP^106–126^. The DLP1 level and mitochondria morphology in N2a cells were determined. Immunobolt results verified that DLP1 protein levels were reduced to <50% of the control level in DLP1 RNAi‐transfected cells (Fig. 3A,B), although the decrease was not statistically significant compared with cells transfected with the scramble sequence (Fig. 3A,B). Two days after transfection, transfected N2a cells were treated with PrP^106–126^ for 12 h before fixation for fluorescence imaging. Compared with the short rod shaped or filamentous mitochondria morphology of control N2a cells, DLP1 RNAi transfection induced mitochondrial elongation (Fig. 3C,D). Suppressed DLP1 expression by RNAi almost completed prevented PrP^106–126^‐induced mitochondrial fragmentation (Fig. 3C,D). The percent of N2a cells with fragmented mitochondria in the DLP1 RNAi group was significantly reduced compared with the PrP^106–126^‐treated, RNAi control group to levels similar to that of the untreated group (Fig. 3E). Fragmented mitochondria were found in DLP1 overexpressed N2a cells, similar to PrP^106–126^ treated, untransfected cells. Taken together, DLP1 overexpression mimics mitochondrial fragmentation in *in vitro* and *in vivo* models of prion diseases and silencing of DLP1 prevents PrP^106–126^‐induced mitochondrial fragmentation, suggesting that DLP1 is a key factor in mitochondrial fragmentation in prion disease.

### Deletion of DLP1 ameliorated PrP^106–126^‐induced mitochondrial dysfunction

After we demonstrated that DLP1 plays a critical role in mitochondria fragmentation, we tested whether DLP1 could also influence PrP^106–126^–induced changes of mitochondrial functions.

**Figure 4:**
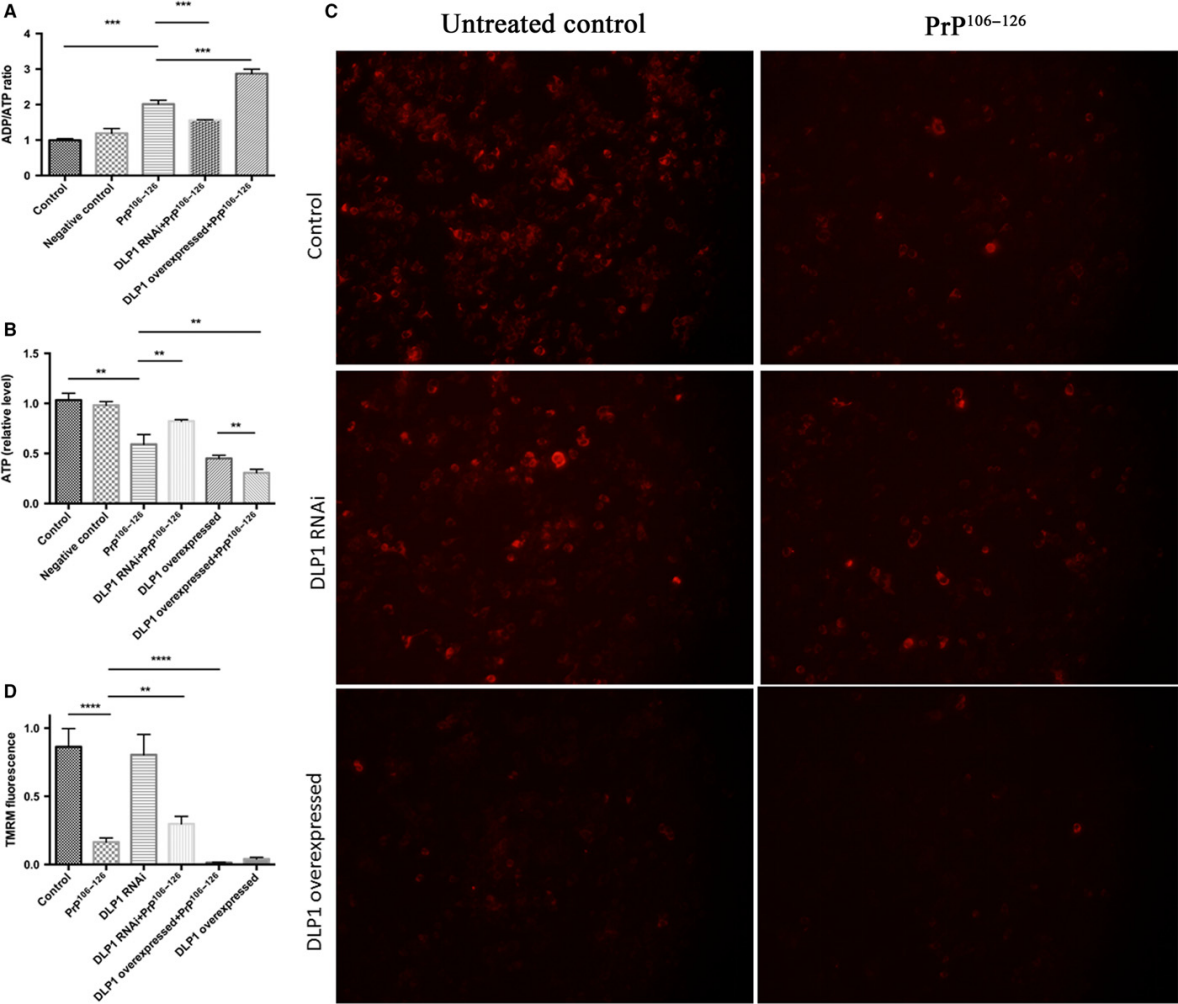
Deletion of DLP1 ameliorated PrP^106–126^‐induced mitochondrial dysfunction. DLP1 RNAi‐transfected, DLP1 overexpressed and control N2a cells were treated with 150 μm PrP^106–126^ for 12 h. Intracellular ADP/ATP ratio (A) and ATP levels (B) were measured using a colorimetric‐fluorometric assay kit, and mitochondrial membrane potential (C, D) was assessed using the potentiometric fluorescent dye, TMRM by fluorescence immunohistochemistry. Experiments were repeated at least three times. Control: wild‐type cells; negative control: cells transfected with scrambled RNAi **P < 0.01, ***P < 0.001, ****P < 0.0001.

Intracellular ADP/ATP ratio and ATP are functional parameters of mitochondrial respiratory chain. ATP levels and ATP/ADP ratio were measured in N2a cells treated with 150 μm PrP^106–126^. The prion peptide caused a rapid increase in ADP/ATP ratio and decrease in ATP level in untransfected cells (Fig. 4A,B). To further investigate the role of DLP1 in PrP^106–126^‐induced bioenergetic changes and mitochondrial fragmentation, DLP1 RNAi or DLP1 overexpressed cells were exposed to the prion peptide and intracellular ATP levels and ADP/ATP ratio were determined. We found that DLP1 knockdown significantly attenuated the increase in ADP/ATP ratio and decrease in ATP level induced by PrP^106–126^ (Fig. 4A,B). On the contrary, overexpression of DLP1 aggravated the effect of PrP^106–126^ on ADP/ATP ratio and ATP level in N2a cells (Fig. 4A,B).

Next we measured mitochondrial membrane potential (MMP), which also is a fundamental marker of mitochondrial function using the potentiometric fluorescent dye, TMRM. PrP^106–126^ treatment caused a markedly decrease in MMP in N2a cells. Decreased TMRM in PrP^106–126^‐treated N2a cells was effectively recovered in DLP1 RNAi cells, while overexpression of DLP1 enhanced the MMP in PrP^106–126–^treated N2a cells (Fig. 4C,D). Taken together, these results suggest, although DLP1 knockdown did not completely restore the PrP^106–126^‐induced mitochondrial dysfunction, it did dramatically ameliorate the impairment of mitochondria.

### Inhibition of DLP1 alleviated neuron apoptosis induced by PrP^106–126^

**Figure 5:**
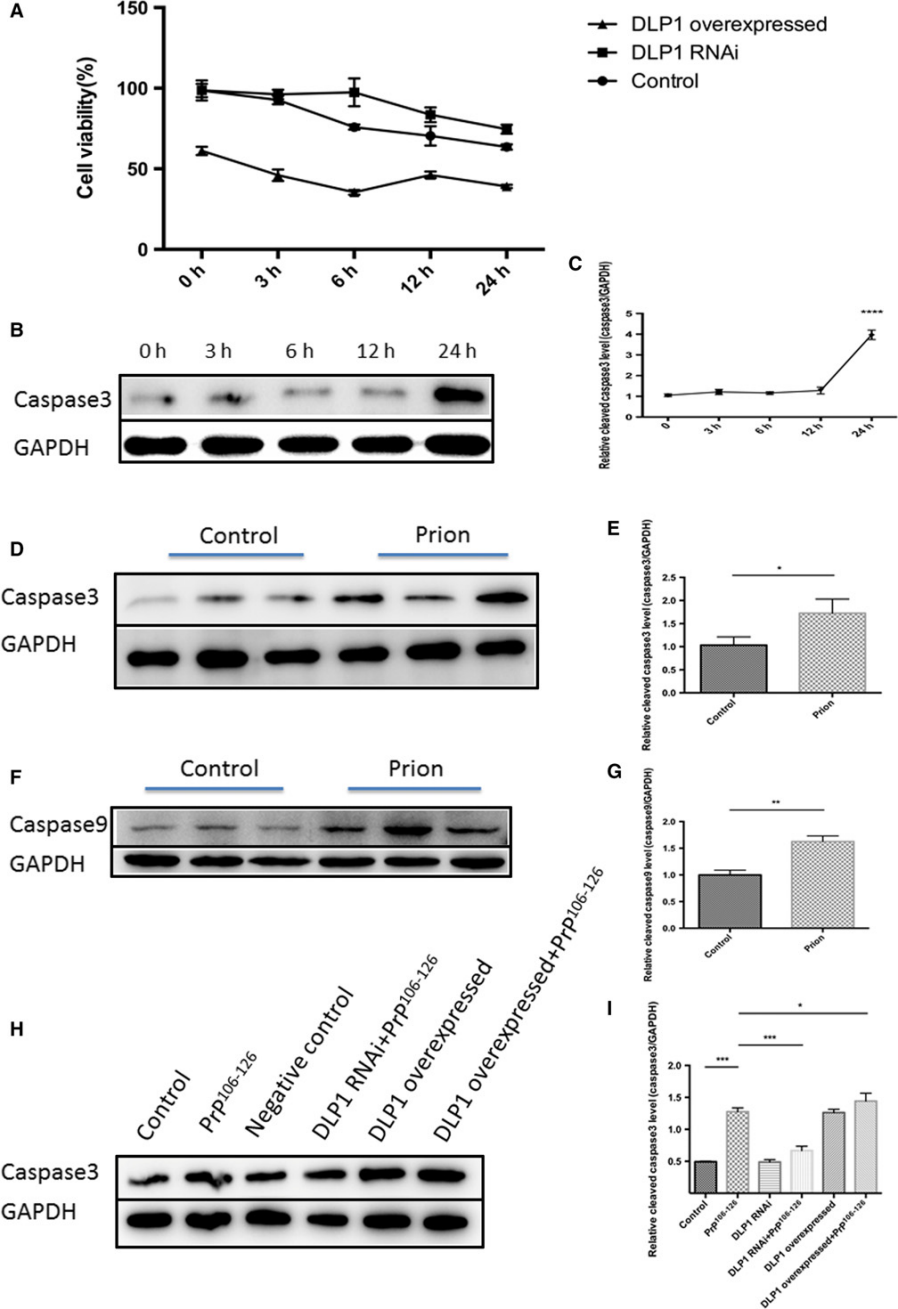
PrP^106–126^‐induced mitochondrial fragmentation resulted in neuronal cell death and apoptosis. Control, DLP1 RNAi, and DLP1 overexpressed N2a cells were treated with 150 μm PrP^106–126^ for up to 24 h, and cell viability was determined by the CCK8 kit (A). Cleaved caspase 3 in untransfected, prion peptide‐treated cells at 0 to 24 h (B, C) was measured by immunoblotting. Cleaved caspase 3 and caspase 9 were increased in prion‐infected hamster brains compared with the age‐matched control (D‐G). Suppression of DLP1 expression in N2a cells inhibited PrP^106–126^‐induced increase in cleaved caspase 3 (a marker of apoptosis) (H–I). Ten prion hamsters and ten age‐matched controls were analyzed. Representative blots are shown. Control: wild‐type cells; negative control: cells transfected with scrambled RNAi. *P < 0.05, **P < 0.01, ***P < 0.001, ****P < 0.0001.

In prions, marked spongiform vacuolation and progressive cell death were two main pathologic changes in neurons. We measured the cell viability and cleaved caspase 3 levels in PrP^106–126^‐treated N2a cells over 24 h. Cell viability started to decrease at 6 h (77.6% of untreated control), and decreased to approximately 60% of control at 24 h after treatment (Fig. 5A). In DLP1 RNAi cells, the cell viability was unaffected for up to 6 h until 12 h when cell viability decreased to 78% of control, indicating suppression of PrP^106–126^‐induced cell death (Fig. 5A). However, DLP1 overexpression significantly decreased cell viability before and after PrP^106–126^ treatment compared with wild‐type and DLP1 RNAi cells.

In PrP^106–126^‐treated cells, cleaved caspase 3 was unchanged for up to 12 h until 24 h when it was markedly increased to approximately fourfold higher than the pretreatment value (Fig. 5B,C). However, mitochondria were fragmented at 12 h after PrP^106–126^ treatment. These findings demonstrated that mitochondrial fragmentation preceded neuronal death and apoptosis. In the terminal‐stage 263K strain‐infected hamsters, the level of cleaved caspase 3 and caspase 9 was also markedly increased (Fig. 5D–G). To determine whether excessive fission underlies PrP^106–126^‐induced neuronal death and apoptosis, we inhibited mitochondrial fission by DLP1 RNAi that blocked mitochondrial fragmentation. DLP1 suppression significantly reduced PrP^106–126^‐induced increase in cleaved caspase 3 (Fig. 5H). In the contrary, overexpression of DLP1 exacerbated cell death and apoptosis as shown by increased level of cleaved caspase 3 (Fig. 5A,H).

Taken together, these findings indicated that the neuron cell death and apoptosis occur *in vitro* and *in vivo* in prion models and the inhibition of mitochondrial fragmentation significantly prevented PrP^106–126^‐induced neuronal death and apoptosis.

### DLP1 participates in regulation of synaptic plasticity and dendritic spines

Dendritic spines are the recipient sites for most excitatory transmissions. Aberrations in dendritic spines were detected in various neurodegenerative and psychiatric diseases manifesting perturbations in cognition and information processing (Yadav *et al*., 2017). PSD95 is a scaffolding protein involved in the assembly and function of the postsynaptic density complex as well as required for spine stability. It is used as an index of synaptic plasticity and spine numbers.

**Figure 6:**
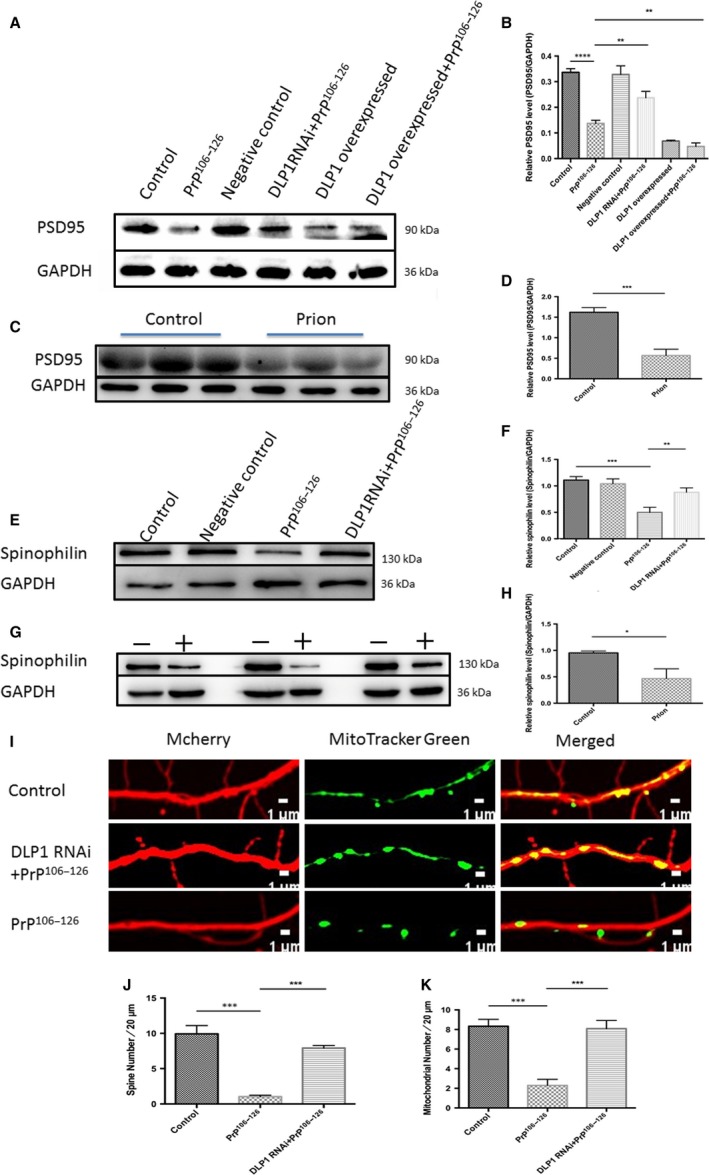
DLP1 is responsible for dendritic spine retraction. Representative immunobolts (A) and quantification analyses (B) showing PSD95 levels in control, negative control, and DLP1 overexpressed groups without treatment with PrP^106–126^ as well as DLP1 RNAi and DLP1 overexpressed groups treated with 150 μm PrP^106–126^ for 12 h in N2a cells. PSD95 was determined in age‐matched control and prion‐infected hamster brains (C‐D). Representative immunobolts (E) and quantification analyses (F) showing spinophilin levels in control, PrP^106–126^, and DLP1 RNAi primary neurons treated with PrP^106–126^. Spinophilin was determined in age‐matched control and prion‐infected hamster brains (G‐H). Representative images of dendrites (I), quantification of dendritic spines (J), and mitochondria puncta (K). Immunostaining was detected by mCherry fluorescence (red) and mitotracker immunostaining (green) in primary rat neurons (DIV14) transfected with DLP1 RNAi. Ten prion hamsters and ten control hamsters were analyzed. All experiments *in vitro* were repeated at least three times. *P < 0.05, **P < 0.01, ***P < 0.001, ****P < 0.0001. Control: wild‐type cells; negative control: cells transfected with scrambled RNAi.

Compared with untreated N2a cells, the level of PSD95 was significantly decreased 12 h after PrP^106–126^ treatment, indicating decreased number of dendritic spines (Fig. 6A,B). Similarly, the level of PSD95 declined slightly in 263K strain‐infected hamster medulla compared with control hamsters (Fig. 6C,D). Suppressed DLP1 expression alleviated the loss of PDS95 in N2a cells treated with PrP^106–126^ (Fig. 6A,B). In contrast, overexpression of DLP1 in PrP^106–126^‐treated N2a accelerated the loss of dendritic spines (Fig. 6A,B).

Spinophilin is another marker of dendritic spine. Immunoblots of spinophilin showed that the level of spinophilin was decreased in both primary neurons *in vitro* and in hamsters *in vivo*, compared with the control group (Fig. 6E–H). Suppressed DLP1 expression in N2a cells prevented the spinophilin reduction induced by the prion peptide, suggesting DLP1 may regulate PrP^106–126^‐induced dendritic spine loss. Furthermore, we monitored the effect of PrP^106–126^ on the density of dendritic spines displayed by primary neurons using immunofluorescence staining. The number of dendritic spines was significantly lower in the PrP^106–126^‐treated primary neurons than the untreated control, and mitochondria in neurites were reduced by almost four fold in PrP^106–126^‐treated primary neurons (Fig. 6I–K). To determine whether these changes correlated with a loss of mitochondria in neural process, DLP1 was overexpressed in primary neurons before exposure to PrP106‐126. We demonstrated that DLP1 RNAi prevented the prion peptide‐induced loss of both mitochondria and dendritic spines in neural processes (Fig. 6I,J). These *in vitro* and *in vivo* findings suggest that dendritic spines are decreased in prion diseases and DLP1 may participate in the regulation of neuronal synaptic plasticity and dendritic spines.

## Discussion

In this study, the crucial and novel finding was that prion‐induced mitochondrial DLP1 excess caused extensive mitochondrial fragmentation and dysfunction as well as neuronal death and decreased synaptic plasticity. These effects on neurons were alleviated by suppression of mitochondrial fission by DLP1 RNAi, suggesting that increased mitochondrial DLP1 may precipitate neuron loss through harmful effect on mitochondrial dynamics and dysfunction.

Firstly, we confirmed altered mitochondrial dynamics *in vitro* in N2a cells and *in vivo* in prion‐infected hamsters. Mitochondria became shortened and fragmented as well as functionally deficient and were redistributed and accumulated in the soma and depleted in neuronal processes in PrP^106–126^‐treated N2a cells and 263K strain‐infected hamster cerebellum and medulla. Intracellular mitochondria distribution is of vital importance to neurons. The morphological complexity and dependence on mitochondria as the energy supply at numerous selective sites of neuronal cells make neurons specifically sensitive to perturbation in mitochondria distribution (Kann & Kovacs, 2007; Wang *et al*., 2009). The absence of mitochondria from axon terminals results in synaptic dysfunction in flies (Stowers *et al*., 2002).

Abundant ATP is necessary for cell metabolism and multiple synaptic functions at nerve terminals such as neurite outgrowth and synaptic plasticity (Fang *et al*., 2016; Manczak *et al*., 2016). After observing that the balance of mitochondrial dynamics is definitely tipped *in vitro* and *in vivo* models of prion diseases, we further showed MMP collapse accompanied by ATP loss in PrP^106–126^‐treated neuron cells *in vitro* and the hamster prion model *in vivo*. The above findings suggest that abnormal mitochondrial dynamics toward enhanced fission might be involved in neuronal dysfunction in prion diseases.

Then, we found that the mitochondrial fission protein DLP1 was markedly and time dependently increased in mitochondria of PrP^106–126^‐treated N2a cells and 263K strain‐infected hamster medulla. In PrP^106–126^‐treated N2a cells, the level of mitochondrial DLP1 rapidly increased on a time‐dependent manner and by 24‐h mitochondrial DLP1 almost tripled levels of untreated N2a cells. In the following studies, we verified prion‐induced excessive mitochondrial fragmentation could be prevented by silencing DLP1, indicating that prion‐associated mitochondrial fragmentation and dynamics was at least partly caused by abnormally increased mitochondrial DLP1.

Interestingly, the cellular level of DLP1 was slightly decreased in prion peptide‐treated N2a cells and 263K strain‐infected hamster brains homogenate, similar to findings in other studies (Yang *et al*., 2016). It is possible that DLP1 is widely distributed in cells (not only in mitochondria but also in cytoplasm). In some pathological conditions including prion diseases, increasing amount of DLP1 is transported from cytoplasm to mitochondria or the degradation pathway of mitochondrial DLP1 is impaired (Wang *et al*., 2016).

Furthermore, in PrP^106–126^‐treated N2a cells and 263K strain‐infected hamster brain homogenate, there was also a dramatic increase in cleaved caspases 3, associated with decreased cell viability in PrP^106–126^‐treated N2a cells. Thus, the above studies strongly demonstrated that not only impairment mitochondrial dynamics but also mitochondrial dysfunction and cell death occur in prion diseases.

In our *in vitro* prion model in N2a cells treated with PrP^106–126^, neuron apoptosis characterized by cleaved caspase 3 was preceded by mitochondrial fragmentation. Thus, we proposed that inhibition of mitochondrial fragmentation by preventing DLP1‐mediated mitochondrial fission impairment might prevent neurodegeneration in prion diseases. Indeed, alleviation of mitochondrial fragmentation by reducing DLP1 expression partly recovered the decrease in MMP and ATP loss, placing mitochondria fragmentation as an upstream event. On the contrary, overexpressing the mitochondrial fission protein DLP1 mimicked the changes in PrP^106–126^‐treated N2a cells, that is, abnormal mitochondrial dysfunction and cell degeneration. Furthermore, the PrP^106–126^ had a deleterious‐additive effect on DLP1 overexpressed cells. That maybe because PrP^106–126^ results in cell apoptosis or death by many different pathways, and DLP1‐mediated mitochondria excessive fission is one of them. Our findings in prion disease models were similar to the neurodegenerative diseases PD and AD, where DLP1 plays a vital role in neuron degeneration (Wang *et al*., 2011, 2016; Kim *et al*., 2016; Manczak *et al*., 2016). Overall, these observations suggest that modulation of DLP1 plays a significant role in the pathogenesis of prion disease.

PrP^Sc^ is often found in neuropil deposits, which are referred to as ‘synaptic‐like’, as they appear to surround synaptic sites (Clinton *et al*., 1993; Belichenko *et al*., 2000; Cunningham *et al*., 2003; Campeau *et al*., 2013). In prion diseases, apart from neuronal loss, earliest and potentially most vital neuropathology changes occur at the level of synapses including synapse loss, morphological changes, and functional abnormality (Mallucci, 2009). Two‐photon imaging studies of living, prion‐infected animals found that retraction of dendritic spines occur early during disease progression (Fuhrmann *et al*., 2007; Fang *et al*., 2016). Changes in their morphology are now believed to underlie synaptic plasticity associated with learning and memory, as well as degenerative events that occur during aging and neurological diseases (Sala & Segal, 2014; Herms & Dorostkar, 2016). These studies pinpointed synapses and dendritic spines as important targets of prion neurotoxicity. However, molecular mechanisms by which prions actually cause synaptic plasticity are still poorly understood.

Mitochondria constantly travel from the cell bodies to nerve terminals to produce and supply sufficient ATP at synapses. We proposed that DLP1 deficiency might alleviate synaptic dysfunction such as neurite loss and synaptic plasticity in prion diseases. The number of dendritic mitochondria affected the number and plasticity of spines and synapses (Li *et al*., 2004). In our prion models, prion treatment decreased spine density in primary neuron cells as well as PSD95, a marker of synaptic plasticity and spine numbers, and prion‐induced mitochondrial fragmentation was accompanied by a decrease in mitochondria coverage in neurites. Partial reduction of DLP1 could efficiently alleviate PrP^106–126^‐induced synaptic loss or dysfunction in primary neurons, suggesting that mitochondrial redistribution and fragmentation mediated by DLP1 probably result in the synaptic dysfunction in prion disease. These data demonstrate that prion‐caused increase in mitochondrial‐DLP1 plays an important role in neurite loss and synaptic abnormalities through modulating mitochondria dynamic balance.

Biochemical and structural studies indicated that the mechanisms of DLP1‐induced mitochondrial fission were similar to those by other members of the dynamin‐superfamily (Richter *et al*., 2015). DLP1 recruited to the outer membrane of mitochondria assembles into a ring structure, which induces the scission of mitochondria. Excessive fission results in mitochondrial fragmentation concomitant with morphological changes and functional loss. Importantly, DLP1‐dependent mitochondrial fission is also required for the segregation of dysfunctional mitochondria and for their specific targeting to mitophagy. Studies demonstrated that suitable mitochondrial fission is required for damaged mitochondria before removal by mitophagy in mouse pancreatic β cells (Twig *et al*., 2008). Besides, downregulation of DLP1 has been reported to prevent mitochondrial autophagy in mouse heart (Ikeda *et al*., 2015; Shirakabe *et al*., 2016). DLP1 has also been described to modulate mitophagy, possibly involving the interaction of DLP1 with the mitophagy receptor FUN14 domain‐containing 1 Pan troglodytes (FUNDC1) (Zuo *et al*., 2014; Wu *et al*., 2016). In yeast, the autophagic scaffold protein atg11p binds to and recruits Dnm1p/DLP1 to promote mitochondrial fission that occurs upon mitophagy (Mao *et al*., 2013). Collectively, these results suggest that DLP1 regulates mitochondrial function by mediation of autophagy and mitophagy. Based on our study findings and previously reported studies, DPL1 might induce mitochondrial fission, fragmentation and mitophagy by interaction with the mitophagy receptor FUN14 and possibly other receptors, which are yet to be elucidated.

Our findings suggest that DLP1 as a critical fission factor promotes neuronal mitochondria fragmentation and impairment of mitochondrial dynamics, and altered mitochondrial dynamics likely contribute to neuronal dysfunction and degeneration in prion diseases. DLP1 has been proposed forward as a treatment target for preventing mitochondrial fragmentation under various pathological conditions (Guo *et al*., 2013; Zhang *et al*., 2013; Filichia *et al*., 2016; Kim *et al*., 2016). The DLP1 mitochondrial fission/fusion machinery as a therapeutic target of prion diseases warrants further study.

## Materials and methods

### Cell culture

Mouse neuroblastoma N2a cells were grown in Dulbecco modified Eagle's medium (DMEM) (Hyclone, Logan, UT, USA) supplemented with 10%(V/V) fetal bovine serum (Gibco, Grand Island, NY, USA) at 37 °C with 5% CO_2_ in a humid incubator. Cerebral cortex neurons were prepared from neonatal rats within 72 h of birth. Cerebral cortex neurons were isolated as described previously (Zhu *et al*., 2016). Briefly, the cortices were digested with DMEM containing 2 mg mL^−1^ papain (Invitrogen, Carlsbad, CA, USA) and 50 mg mL^−1^ DNase (Sigma‐Aldrich, St. Louis, MO, USA) for 50 min at 37 °C. Digested tissues were filtered with a 70 μm nylon cell strainer (Corning, Corning, NY, USA). Isolated cells were seeded in 0.05 mg mL^−1^ poly‐L‐lysine (Solarbio, Beijing, China)‐coated plates at a density of 2 × 10^5^ cells per well in a 24‐well plate. Neurons were cultured in DMEM/F12 (Gibco) containing 1% B27 serum‐free supplement (Invitrogen), and 1% penicillin/streptomycin (Gibco). After 48 h, 10 mm cytarabine (Sigma‐Aldrich) was added to suppress the growth of glial cells. Experimental treatments were performed after 7 days of culture.

### Transfection and infection

Primary neurons and N2a cells were transfected with plasmid using the Lipofectamine 2000 reagent (Invitrogen). Plasmid DNA (1 mg) was added to 1 × 10^5^ adherent cells in a 24‐well plate and incubated with in 3 μL of Lipofectamine 2000 and 75 μL Opti‐MEM medium for 5 min at room temperature.

A mCherry‐encoding lentivirus (created using the vector H4446 pLenti‐hsyn‐mcherry) was obtained from the OBiO company. Virus (1 × 10^5^ TU mL^−1^ final concentration) was added to hippocampal neurons after 7 days in culture. Neurons were then cultured for an additional 3 days, to maximize mCherry expression, before PrP^106–126^ treatment.

### Prion protein peptide

PrP^106–126^ peptide of >95% purify (sequence KTNMKHMAGAAAA GAVVGGLG) was synthesized by Sangon Bio‐Tech (Shanghai, China). The peptide was dissolved in 0.1 m phosphate buffer saline (PBS) to a concentration of 1 mm and shaken at 4 °C for 24 h to let the peptide aggregate. All the procedures were performed under sterile conditions. Experiments were conducted with a final peptide concentration of 150 μm.

### Expression vectors, antibodies, chemicals, and measurement

PCMV‐HA plasmid (Invitrogen), Mito‐GFP construct (Clontech, Mountain View, CA USA), pEGFP‐N1 (Clontech). The DLP1 RNAi sequence targeting the open reading frame region of rat and mouse DLP1 was described previously (Wang *et al*., 2011) and the negative control containing a scramble sequence with no significant similarity to mouse, rat, or human gene sequences were obtained from GenePharma, Shanghai. The sequence of RNAi is presented in Table 1. The DLP1 primers containing restriction sites SalI and XholI were (5′ GTCGACCGCCACCATGGAGGCGCTGATCCCGGT 3′) and (5′ CTCGAGTCACCAAAGATGAGTCTCTC 3′), respectively. Primary antibodies include rabbit anti‐DLP1 (Cell Signaling Technology), rabbit anti‐caspase3 (Abcam, Cambridge, MA USA), rabbit anti‐caspase9 (Abcam, China), rabbit anti‐PSD95 (Proteintech, Mountain View, CA USA), rabbit anti‐spinophilin (Cell Signaling Technology, Boston, MA USA), mouse anti‐COX IV (Cell Signaling Technology), mouse anti‐GAPDH (Sigma‐Aldrich), HRP‐goat anti‐mouse (Sigma‐Aldrich), HRP‐goat anti‐rabbit (Sigma‐Aldrich), and 594 goat anti‐rabbit (Sigma‐Aldrich). Tetramethylrhodamine methyl ester (TMRM) for the measurement of mitochondrial membrane potential was obtained from Sigma‐Aldrich. ATP level and ADP/ATP radio were measured by the Colorimetric‐Fluorometric Assay Kit (Biovision, Milpitas, CA, USA). The Mitotracker@green was purchased from Life Technologies American.

**Table 1:** RNAi sequence targeting the open reading frame region of rat and mouse DLP1 and the negative control sequence

Name	Sense	Antisense
DLP1 RNAi	5′AACCCUUCCCAUCAAUACAUC3′	5′GAUGUAUUGAUGGGAAGGGUU3′
Negative control	5′UUCUCCUGAACGUGUCACGUTT3′	5′ACGUGACACGUUCGGAGAATT3′

### Prion hamster brain samples

Twenty Syrian golden hamsters of 6 weeks old were obtained from the Vital River Company (USA) and divided into two groups. One group was infected with the 263K scrapie strains, and the other was age‐matched controls. The hamsters were intracerebrally inoculated with 6–8 μL of 10% (w/v) brain homogenate in 0.01 m phosphate‐buffered saline from either a normal brain or a 263K‐infected hamster brain at the terminal stage of the disease. When the clinical signs of prion disease were observed in the late‐stage, the hamsters were sacrificed.

### Mitochondrial isolation

All procedures for purified mitochondrial isolation were conducted at 4 °C. Briefly, cells and freshly prepared tissue were washed in 0.9(w/v) sodium chloride solution. The cells or tissue was homogenized with lysis buffer (Beyotime, Shanghai China) supplemented with a protease inhibitor solution (Beyotime) and then centrifuged at 1000 *g* for 10 min to remove nuclear contaminants, cell debris, and intact cells. The supernatant was transferred to a clean 1.5‐mL tube and centrifuged again at 6000 *g* for 10 min at 4 °C. The supernatant constituting the microsomal fraction was removed.

### Examination of mitochondria by immunohistochemistry

Hamster brain tissue was fixed for 24 h before 6‐μm‐thick slices were consecutively sectioned as described previously (Zhu *et al*., 2000). Ten prion and ten control animals were sacrificed in this experiment. Immunohistochemistry staining of the brain sections for mitochondria using anti‐COX IV antibodies was performed according to the protocol as described previously by Zhu *et al*. (2000). The stained tissue sections were examined under an Olympus microscope.

### Western blotting

Western blotting was performed by loading 40 μg proteins in each lane on 12% SDS‐PAGE gels. Nonspecific binding sites of the nitrocellulose membrane were blocked using 5% BSA skinned milk (BD, Franklin Lakes, NJ USA). The blots were imaged using the ChemiDoc Imaging System (Bio‐Rad, Hercules, California, USA).

### Immunofluorescence microscopy and quantitative analysis of mitochondria and dendritic spine

N2a and primary neuronal cells were seeded on 24‐well culture plates. After treatment with PrP^106–126^ or control peptide, the cells were fixed and stained as described previously (Liu *et al*., 2016; Zhu *et al*., 2016). An Olympus confocal microscope was used for capturing fluorescence images. For each neuron, dendritic segments 100–200 μm in length beginning 100 μm from the cell body were selected. Most mitochondria were well separated in binary images, and large clusters of mitochondria were excluded automatically. All binary images were analyzed by ImageJ for mitochondria length. Mitochondria were also manually counted and length measured by an experienced pathologist in a blinded manner.

### Cell viability assay

Cell Counting Kit‐8 (Beyotime) was used to measure cell viability. After treatment, the CCK‐8 solution was directly added to cell culture medium before incubation for 30 min at 37 °C and 5% CO_2_. The absorbance at 450 nm was obtained using a microplate reader with a background control as the blank. The cell viability was expressed as the percentage of the untreated control.

### Transmission electron microscopy

The prion and control group hamster brains were cut into small pieces after fixation in 5% glutaraldehyde in 0.1‐m sodium cacodylate buffer (pH 7.4) for 4 h at 4 °C. After rinsing three times with PBS, the tissue was further fixed in 1% OsO4 in 0.1‐m sodium cacodylate buffer for 2 h before three washes with PBS. Double fixed tissue pieces were dehydrated with a series of ethanol and acetone and then were embedded in resin before polymerization at 60 °C for 48 h. Ultrathin sections were mounted onto copper grids and stained with 4% uranyl acetate and lead citrate. Imaging was performed using a transmission electron microscope (JEM‐1230, Tokyo, Japan) operating at 80 or 120 kV.

### Statistical analysis

All assays were repeated three times. Data were expressed as means ± SD. Parametric data were analyzed using Student's *t*‐test or one‐way ANOVA followed by post hoc Turkey's test using the spss software (version 13.0: SPSS Inc., Chicago, IL, USA), GraphPad Prism 5 software (La Jolla, CA, USA), or Image J (National Institutes of Health, Bethesda, MD, USA). *P* < 0.05 was considered statistically significant.

## Funding

This work was supported by the Natural Science Foundation of China (Project No. 31472166), Ministry of Agriculture of China, 948 projects (2014‐S9) and Chinese Universities Scientific Fund (Project No. 2017DY003).

## Conflict of interest

None declared.
